# Inadequate Response, Treatment Patterns, Health Care Utilization, and Associated Costs in Patients With Ulcerative Colitis: Retrospective Cohort Study Based on German Claims Data

**DOI:** 10.1093/ibd/izab330

**Published:** 2022-02-04

**Authors:** Bernd Bokemeyer, Nils Picker, Thomas Wilke, Ludger Rosin, Haridarshan Patel

**Affiliations:** Interdisciplinary Crohn Colitis Centre Minden, Minden, Germany; Ingress Health HWM GmbH, Wismar, Germany; IPAM e.V., Wismar, Germany; Galapagos Biopharma Deutschland GmbH, München, Germany; Galapagos NV, Mechelen, Belgium

**Keywords:** ulcerative colitis, real-world treatment, suboptimal therapy, advanced therapy, steroids

## Abstract

**Background:**

Real-world data regarding response rates in ulcerative colitis treatment are rare, particularly for later lines of therapy. This study aimed to assess continuity of and changes to advanced therapies, as well as costs and specific indicators defining suboptimal therapy.

**Methods:**

German claims data were retrospectively analyzed (January 2014 to June 2019). Patients with ulcerative colitis initiating an advanced therapy (adalimumab, golimumab, infliximab, tofacitinib, vedolizumab) were included. Inadequate response was indicated by therapy discontinuation, switch, escalation, augmentation, corticosteroid dependency, disease-related hospitalization, or surgery. Health care resource utilization (inpatient, outpatient, sick leaves, medication, aids, and remedies) and related costs were assessed from therapy initiation until discontinuation or loss to follow-up.

**Results:**

Among 574 patients (median age, 39 years; female sex, 53.5%) who initiated advanced therapies, 458 (79.8%) received an antitumor necrosis factor therapy, 113 (19.7%) vedolizumab, and 3 (0.5%) tofacitinib. After 12 months, 75% had ≥1 indicator for suboptimal therapy. The median time to first indicated inadequate response was 4.8 months. Therapy discontinuation (38%), switching (26%), and prolonged use of steroids (36%) were common within the first year of treatment. In an unadjusted comparison, all-cause total costs per person-year were significantly higher in those who switched vs patients remaining on their therapy (€44,570 vs €36,807; *P* < .001).

**Conclusions:**

Our study indicates a high prevalence of inadequate response to advanced therapies. Only 25% of patients showed adequate response within 12 months after therapy initiation. Frequent dose and treatment changes were observed. The economic impact of suboptimal therapy in ulcerative colitis is substantial, highlighting the ongoing need for improved treatment strategies.

Inflammatory bowel disease (IBD), comprising Crohn’s disease (CD) and ulcerative colitis (UC), is characterized by chronic inflammation in the gastrointestinal tract but can also come with extraintestinal manifestations. The IBD-related disease burden is considerably high, with an estimated number of approximately 6.8 million cases of IBD in 2017 worldwide.^1^ In Germany, there are about 320 000 men and women living with IBD.^2^ Approximately half of the IBD patients have UC as underlying pathology, whereas the other half of IBD patients are diagnosed with CD.^2, 3^ Patients with moderate to severe UC show impaired quality of life, loss of work productivity, and experience limitations to engage in social activities.^4, 5^

The overall treatment goal in UC patients is disease remission in conjunction with a good quality of life without requiring long-lasting corticosteroid (CS) use.^6, 7^ Pharmaceutical treatment options include aminosalicylates, conventional immunosuppressants, and advanced/biological therapies.^7^ Patients with moderate to severe UC for whom conventional therapy failed are recommended to start a second line of treatment with antitumor necrosis factor α (anti-TNFα) agents such as infliximab, adalimumab, and golimumab, or the anti-integrin antibody vedolizumab.^8^ More recently, the Janus kinase (JAK) inhibitor tofacitinib and additionally the interleukin-12/23 inhibitor ustekinumab have become available as a treatment of UC. In addition, a new generation of JAK inihibitors (filgotinib and upadacitinib) is currently in clinical development, offering another alternative for the treatment of UC in the near future.

Patients with successful response after induction therapy are recommended maintenance therapy to retain remission in the long-term. However, real-world data on treatment of UC patients with biologic and other advanced therapies in Germany remains sparse. Recent German claims analyses on treatment patterns in patients with IBD showed that residual disease during ongoing treatment and advanced therapy discontinuation were common.^9,10^ However, no studies were conducted regarding patients with UC in specific. Furthermore, there is a lack of current data on recently approved advanced therapies for UC, such as tofacitinib. Therefore, this study primarily aimed to describe the rates of inadequate response to advanced therapies in patients with UC. In addition, we examined the frequency of serious adverse events (SAEs), health care resource utilization (HCRU), and associated costs in patients with UC treated with advanced therapies.

## Materials and Methods

### Data Set

This was a retrospective cohort study utilizing data from a regional German statutory health insurance (AOK PLUS) that insured approximately 3.4 million individuals in the German states Saxony and Thuringia. The data include anonymized patient-level information on sociodemographic characteristics, outpatient drug prescriptions, inpatient and outpatient diagnoses and treatments, HCRU and associated costs, and other information such as days absent from work.

### Sample Selection

Generally, the study population comprised patients aged 18 years or older with a UC diagnosis between January 1, 2014, and June 30, 2019, who were continuously insured during the entire baseline period (from January 1, 2014, until first diagnosis for UC). The presence of UC was considered if the patient had at least 1 inpatient diagnosis (ICD-10 code K51.-) and/or 2 outpatient diagnoses of UC recorded in 2 different quarters. Patients were included in the analysis if they initiated a UC-related treatment with an advanced therapy between January 1, 2015, and June 30, 2019. To ensure the inclusion of patients who had newly started treatment with an advanced agent, a minimum pre-index period of 12 months (baseline period) without prescription of the index therapy was defined. The following agents were considered as advanced therapy for management of UC and were identified by the corresponding Anatomical Therapeutic Chemical (ATC) codes and operational and procedure (OPS) codes (Supplemental Table 1): adalimumab, golimumab, infliximab, tofacitinib, and vedolizumab. No OPS code was available for tofacitinib, hindering the identification of inpatient treatment with tofacitinib.

The date on which a patient initiated an advanced therapy was considered the start of follow-up (ie, the index date). The study end points were investigated for patient-individual follow-up periods ending at death, end of insurance with AOK PLUS, or the June 30, 2019 (whichever came first).

Patients who received prescriptions of more than 1 advanced therapy at the index date were excluded from the study. Other exclusion criteria were a concomitant diagnosis of CD (ICD-10 K50.-) or colitis indeterminate (ICD-10 K52.3) within the 12-month pre-index period, but only if 1 of these diagnoses was the last diagnosis made before the index date (ie, no subsequent UC diagnosis before the start of the advanced therapy). Furthermore, patients with a concomitant diagnosis of rheumatoid arthritis, psoriasis, multiple sclerosis, ankylosing spondylitis, psoriatic arthritis, hidradenitis suppurativa, uveitis intermedia, uveitis posterior and panuveitis were excluded from the analysis, ensuring that UC was causing the start of the advanced therapy and not another concomitant indication (Supplemental Figure 1).

### End Points

Inadequate response was assessed using proxies, which have been previously used by Patel et al.^11^ Accordingly, an inadequate response was assumed when therapy discontinuation, therapy switch, dose escalation, augmentation with conventional therapies, prolonged use of CS, a UC-related hospitalization, or a UC-related surgery had been observed. The earliest identification of one of these proxies was defined as the time of the first observed inadequate response.

A treatment was assumed to be discontinued if there was a prescription gap of >60 days after consuming the previous prescription’s supply. The information on the days of supply was derived by the OPS codes, only specified for infliximab and adalimumab. In case of golimumab (28 defined daily doses [DDDs]) and vedolizumab (56 DDDs), standardized treatment schedules for therapy maintenance were assumed. Information on the days of supply was also derived by the DDD as specified for each ATC code by WIdO/WHO. The date of discontinuation of the therapy was assumed to be the first day of such a 60-day gap. Treatment switching was supposed when a patient received a prescription of another advanced agent without a refill of the index agent within 180 days after the new advanced agent was prescribed.

A dose escalation was assumed if there was a dose increase of the index therapy in the maintenance phase of >1.5 times the recommended dosage according to European Summaries of Product Characteristics (SmPC) over the course of 3 consecutive prescription intervals. Dose escalation was calculated over a minimum follow-up period of 6 months. Start of therapy maintenance was set to 4 weeks after starting adalimumab, 6 weeks after starting golimumab, 14 weeks after starting infliximab or vedolizumab, and 8 weeks after starting tofacitinib.

Augmentation was defined as an incident prescription of a UC-associated conventional therapy that was not active at the index date (prescribed in the recent 3 months before the index date with a DDD-based supply plus 15% grace supply that would cover the index date itself). Conventional therapies included systemic and local acting CS, budesonide (oral and rectal formulation), 5-aminosalicylic acid (5-ASA; oral and rectal formulation), and conventional immunosuppressives (azathioprine, mercaptopurine, methotrexate, ciclosporin or tacrolimus).

Prolonged use of CS was assumed if at least 2 outpatient CS prescriptions had been observed during the observable follow-up period after the index date.

Ulcerative colitis–related hospitalizations were defined by any inpatient admission with a primary diagnosis of UC (ICD-10 code: K51.-) or UC-related complications. Ulcerative colitis–related surgeries were identified by respective inpatient and outpatient procedure codes (codes used are provided in Supplemental Table 1).

Additional end points included HCRU and SAEs leading to hospitalizations. HCRU was assessed in terms of the number of inpatient stays and related hospitalization days, the number of days absent from work, and the number of outpatient visits ordered by general practitioners (GPs), gastroenterologists, and other specialists, approximated by counted dates of invoiced codes according to the uniform evaluation scheme (Einheitlicher Bewertungsmaßstab [EBM] code). An item was defined as a UC-related HCRU if the claim was directly related to UC (ie, hospitalizations with UC as main diagnosis, outpatient visits with a documented UC diagnosis, and days absent from work due to UC). Regarding UC-related hospitalizations, the frequency of specific surgeries (ileal pouch anal anastomosis [IPAA; OPS 5-45], rectal procedure [OPS 5-46], anal procedure [OPS 5-48], and others [OPS 5-49]) was separately reported. Serious adverse events related to UC were assessed as events leading to hospital admission based on the documented main diagnosis code (ICD-GM-10) during the induction phase (first 90 days after start with an advanced therapy) and the maintenance phase (starting at 90 days after index date). Only the most frequent events are reported in this article, including anemia, primary infections, opportunistic infections, skin conditions, and rectal or anal abscesses.

Cost ratios were calculated separately for outpatient care, inpatient care, outpatient medications, remedies and aids, and sick leaves (indirect costs) during 2015-2019 without adjustment for inflation. The reimbursement of services in the outpatient care setting in Germany is regulated by a uniform evaluation standard (EBM), and thus, services are not invoiced directly by means of monetary value but by a system of weighted points. To assess the costs of outpatient care, the weighted points were multiplied by a uniform orientation value, which is defined by the National Association of Statutory Health Insurance Physicians. Inpatient costs covering all performed services and administered drugs during inpatient stays were determined according to the German Diagnosis Related Groups (G-DRG) system. Medication costs were calculated based on outpatient drug prescriptions and respective pharmacy selling prices, as relevant at the respective prescription times. Aids (devices to support the patient) and remedies (ie, services provided by medically trained personal, eg, massages, physiotherapy) were assessed based on the expenses covered by the sickness fund. Indirect costs due to sick leave days were calculated by multiplying the number of sick leave days by the mean daily loss of productivity for a working person in Germany per calendar year according to the Federal Institute for Occupational Safety and Health (BAuA^12^).

### Subgroups

Indicators for inadequate response were additionally investigated in the subgroups of bio-naïve and bio-experienced patients prior to indexand of patients with and without a prolonged use of CS (≥2 prescriptions during the follow-up period). Bio-naïve patients were defined as patients who did not receive any prescription of an advanced therapy (ATC codes L04AA/L04AB) in the 12 months before the index period. Bio-experienced patients did not receive the index agent in the 12 months before the index period. Health care resource utilization and costs were separately described for patients who maintained the advanced therapy compared with those who switched their index therapy during the observable follow-up period, as well as for patients with and without therapy escalation.

### Statistical Analyses

Patient characteristics at baseline were analyzed by using descriptive statistics for the overall population and the predefined subgroups. Absolute and relative frequency tabulations described categorical variables such as sex, age category, insurance status, index therapy, and previous CS use. Means and standard deviations (SD)s were reported for continuous variables such as age at index and Charlson Comorbidity Index (CCI)^13^ based on diagnosis identified in the 12 months before the index period. Comparisons between subgroups were made by using the χ^2^ test statistic for categorical variables, independent *t* test (or the Welch test) for continuous variables. Cost ratios were compared using confidence intervals based on estimated standard errors from a bootstrap analysis with 1000 replications.

The proportion of patients with an inadequate response (composite rate and separately for each defined proxy) to the index therapy was assessed after 3, 6, 9, 12, and 24 months. Furthermore, the time to inadequate response was investigated by means of Kaplan-Meier curves, reporting the median time to event and the interquartile range (IQR) for the overall population and the subgroups. Comparisons between subgroups were made by using the log-rank test. Additionally, the time to therapy switch (as an important component of the inadequate response composite proxy) and respective patterns regarding subsequent treatment lines have been evaluated by using Kaplan-Meier estimation and a Sankey diagram.

A Cox proportional hazards model was used to estimate the relationship between patient baseline characteristics and time to inadequate response to the index therapy. The model estimating the time to inadequate response included the covariates age, gender, index therapy, year of index therapy initiation, bio-experience prior to index, use of CS and other immunosuppressive drugs that were active at the start of index therapy, and prior hospitalization or surgery related to UC. For each covariate, the impact on the time to inadequate response had been evaluated based on the estimated hazard ratio (HR) and related 95% confidence interval (CI).

Health care resource utilization and associated cost ratios were calculated for a follow-up period of up to 12 months and were reported per observed patient year. In case of treatment discontinuation or change of index therapy, patient follow-up was censored at 90 days after the last prescription for the index therapy.

### Regulatory Aspects and General Considerations

Because the study addressed a retrospective anonymized data set, no ethical review and no informed consent of patients were needed. The study protocol was reviewed and approved by a scientific steering committee consisting of experts from the sickness fund, the study sponsor, the research organization conducting the analysis, and independent clinical experts.

Data management was carried out by using SQL (Microsoft SQL Server 2008R2), and statistical analyses were done in Stata version 16.1 (StataCorp LP, College Station, TX).

## Results

A total of 19 135 patients with UC were identified from the claims database, of which 1343 (7.0%) patients were 18 years of age or older and had received an advanced agent within the study period. Continuous insurance during the study period could not be ascertained for 323 patients, 202 patients received the identified index advanced therapy prior to the index date (and thus were not classified as therapy starter), and 244 study exclusions were due to other underlying conditions. The final study population included 574 patients with UC starting a new advanced therapy (Supplemental Figure 2).

### Patient Baseline Characteristics

The mean age at study inclusion was 41.9 years (SD, 15.6). There were 307 (53.5%) female patients, and 267 (46.5%) male patients. The mean Charlson Comorbidity Index without age factor was 1.0 (SD, 1.7). A total of 502 (87.5%) patients were bio-naïve prior to the index therapy, whereas 72 (12.4%) patients were experienced with advanced therapies (all 72 were previously treated with anti-TNF). Of those that were bio-experienced, 48 (66.7%) patients had been on 1 biologic therapy, and 24 (33.3%) patients had been on ≥2 therapies prior to the index therapy. The majority of included patients had used CS prior to the index date (*n* = 496, 86.4%), but prolonged CS use during index therapy was identified in less than half of the patients (*n* = 252, 43.9%). Patients with concomitant CS use were generally older (43.7 vs 40.4 years, *P* = .044) and had a higher CCI (mean CCI without the age factor 1.09 vs 0.91, *P* = .051; Table 1).

**Table 1.** Overview of patient characteristics at baseline.

	Overall	Advanced Therapy Prior to Index Therapy			No. Advanced Therapies Prior to the Index Therapy			With Prolonged Use of CS During Follow-up		
		Bio-naïve	Bio-experienced	*P* ∗	1 advanced therapy	≥2 advanced therapy	*P* ∗	No	Yes	*P* ∗
*n* (no. patients)	574 (100%)	502 (87.5%)	72 (12.4%)		48 (66.7%)	24 (33.3%)		322 (56.1%)	252 (43.9%)	
Mean age at index (SD)	41.9 (15.6)	42.1 (15.8)	40.6 (14.3)	0.521	41.1 (15.2)	39.7 (12.6)	1.000	40.4 (14.6)	43.7 (16.6)	0.044
*Age groups, n (%)*										
18-34	237 (41.3%)	204 (40.6%)	33 (45.8%)	0.394	22 (45.8%)	11 (45.8%)	0.627	138 (42.9%)	99 (39.3%)	0.163
35-54	204 (35.5%)	176 (35.3%)	28 (37.3%)		17 (35.4%)	11 (45.8%)		119 (37.0%)	85 (33.7%)	
55-74	116 (20.2%)	106 (21.1%)	10 (13.9%)		8 (16.7%)	2 (8.3%)		59 (18.3%)	57 (22.6%)	
75-84	17 (3.0%)	16 (3.2%)	1 (1.3%)		1 (2.1%)	0 (-)		6. (1.9%)	11 (4.4%)	
≥85	0 (-)	0 (-)	0 (-)		0 (-)	0 (-)		0 (-)	0 (-)	
Gender, *n* (%)										
Males	267 (46.5%)	229 (45.6%)	38 (54.4%)	0.255	27 (56.3%)	11 (45.8%)	0.404	137 (42.6%)	130 (51.6%)	0.031
Females	307 (53.5%)	270 (54.4%)	34 (47.2%)		21 (43.8%)	13 (54.2%)		185 (57.5%)	122 (48.4%)	
*Insurance status at index, n (%)*										
Employed	477 (83.1%)	416 (82.9%)	61 (84.7%)	0.838	41 (85.4%)	20 (83.3%)	0.901	278 (86.3%)	199 (79.0%)	0.151
Pensioner	63 (12.2%)	56 (11.2%)	7 (9.7%)		5 (10.4%)	2 (8.3%)		29 (9.0%)	34 (13.5%)	
Unemployed	19 (3.5%)	17 (3.4%)	2 (2.8%)		1 (2.1%)	1 (4.2%)		9 (2.8%)	10 (4.0%)	
Voluntarily insured	10 (1.7%)	8 (1.6%)	2 (2.8%)		1 (2.1%)	1 (4.2%)		5 (1.6%)	5 (2.0%)	
Family-insured	5 (0.9%)	5 (1.0%)	0 (-)		0 (-)	0 (-)		1 (0.3%)	4 (1.6%)	
mean CCI without age factor (SD)	1.0 (1.7)	0.99 (1.69)	1.01 (1.96)	0.674	1.15 (2.12)	0.75 (1.59)	0.3188	0.91 (1.69)	1.09 (1.77)	0.051
Previous CS usage, *n* (%)	496 (86.4)	437 (87.0%)	59 (82.0%)	0.237	39 (81.3%)	20 (83.3%)	0.828	268 (83.2%)	228 (90.5%)	0.012

Patient baseline characteristics of the overall sample of patients initiating an advanced therapy for management of UC and of predefined subsamples with and without prior experience to advanced therapies/CS dependency (at least 2 prescriptions while on index therapy). All characteristics refer to either index date (age, gender, insurance status) or a baseline period of 12 months (CCI, previous CS usage). Abbreviations: CCI, Charlson Comorbidity Index; CS, corticosteroids.

Differences between subgroups were assessed by χ^2^ test for categorical variables or by the Welch *t* test for continuous variables.

### Inadequate Response to Advanced Therapies

**Figure 1.**
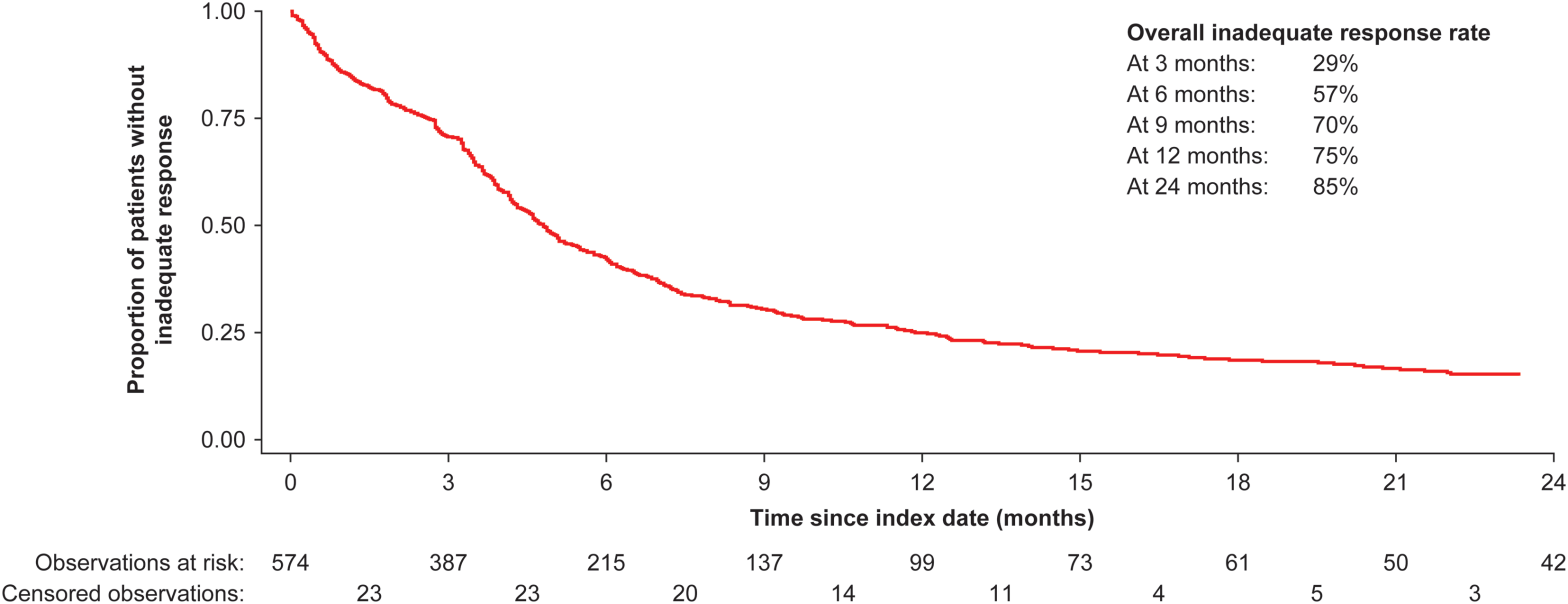
Time to inadequate response among patients with UC treated with advanced therapy.

**Figure 2.**
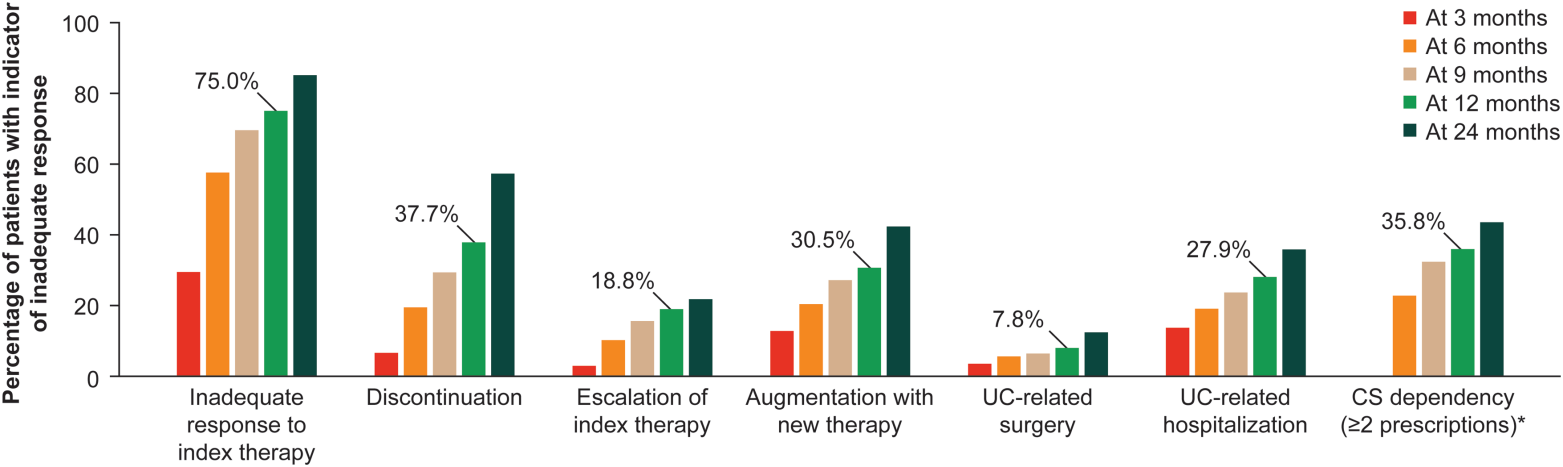
Inadequate response to index therapy by indicator measured at 3, 6, 9, 12, and 24 months. ∗Only prescriptions during maintenance therapy (>14 weeks after index date) were considered in this analysis. Abbreviations: CS, corticosteroids; UC, ulcerative colitis.

The inadequate response rate was 75% (*n *= 395) and 85% (*n *= 429) at 12 and 24 months of follow-up, respectively. The median time to inadequate response of the index therapy in UC patients was 4.8 months (IQR, 2.6-11.9 months; Figure 1). At 12 months, 185 patients had discontinued or switched therapy (37.7%), and 59 patients had experienced a dose escalation (18.8% with dose >150% of SmPCs). For these patients, the average daily dose prescribed during the maintenance therapy was 5.3 mg for adalimumab (186% of recommended maintenance dose), 14.6 mg for infliximab (212% of recommended maintenance dose for patients weighing 77 kg), 3.7 mg for golimumab (207% of recommended maintenance dose for patients weighing <80 kg), and 11.1 mg for vedolizumab (207% of recommended maintenance dose). Due to lack of sufficient number of observations, a maintenance dose could not be calculated for users of tofacitinib. One hundred thirty-nine patients had a treatment augmentation (30.5%), 35 patients had a UC-related surgery (7.8%), 133 patients had a UC-related hospitalization (27.9%), and 139 patients were in need of a prolonged use of steroids while receiving maintenance therapy with an advanced agent (35.8%; Figure 2).

Likelihood of experiencing an inadequate response to the index therapy was higher for patients concomitantly using CS at the start of the index therapy (HR, 1.28; 95% CI, 1.03-1.59) and for patients that were previously hospitalized due to UC (HR, 1.60; 95% CI, 1.27-2.02). With a HR of 0.73 (95% CI, 0.59-0.90), patients who were on other conventional therapies at the start of the index therapy were less likely to experience an inadequate response compared with patients that did not receive a conventional therapy at index. By design, fewer augmentations were observable in patients with initial concomitant use of conventional therapies (Table 2). Bio-experienced patients were not associated with an earlier inadequate response (*P* = .165). However, patients with previous exposure to 2 or more biologics were at higher risk for inadequate response compared with patients who had received only 1 prior agent (Supplemental Figure 3).

**Table 2.** Multivariate Cox regression model estimating factors associated with the probability to indicators of time to first inadequate response to advanced therapies in patients with UC.

Covariate	HR (95% CI)	*P*
Age ≥40 years (*n* = 274)	1.09 (0.89-1.34)	0.394
Female sex (*n* = 305)	0.83 (0.68-1.01)	0.059
**Index therapy**		
Adalimumab (*n* = 230)	Reference	NA
Infliximab (*n* = 172)	1.15 (0.92-1.44)	0.231
Golimumab (*n* = 72)	1.11 (0.80-1.55)	0.533
Vedolizumab (*n* = 113)	0.70 (0.53-0.93)	0.013
**Year of Index**		
2015 (*n* = 142)	Reference	
2016 (*n* = 100)	0.86 (0.64-1.13)	0.278
2017 (*n* = 102)	1.07 (0.80-1.42)	0.644
2018 (*n* = 159)	0.85 (0.65-1.12)	0.246
2019 (*n* = 68)	1.08 (0.68-1.72)	0.743
CCI	1.19 (0.97-1.45)	0.088
**Prior advanced therapy**		
Yes (*n* = 499)	1.18 (0.93-1.50)	0.165
**Active CS use at index date**		
Yes(*n* = 389)	1.28 (1.03-1.59)	0.024
**Other active drugs for UC at index date**		
Yes (*n* = 362)	0.73 (0.59-0.90)	0.004
**Prior UC hospitalization**		
Yes (*n* = 126)	1.60 (1.27-2.02)	<0.001
**Prior UC surgery**		
Yes (*n* = 32)	0.97 (0.65-1.47)	0.901

Multivariate Cox regression model estimating factors associated with the probability to observe indicators of inadequate response in all UC patients treated with biologics (*n* = 571). Patients receiving tofacitinib were excluded due to small sample size (*n* = 3). Other drugs active at index date included 5-aminosalicylic acid (*n* = 359), ciclosporin (*n* = 1), methotrexate (*n* = 1), tacrolimus (*n* = 3), and thiopurines (*n* = 94). Abbreviations: CCI, Charlson Comorbidity Index; CS, corticosteroids; HR, hazard ratio; UC, ulcerative colitis.

### Treatment Patterns

**Figure 3.**
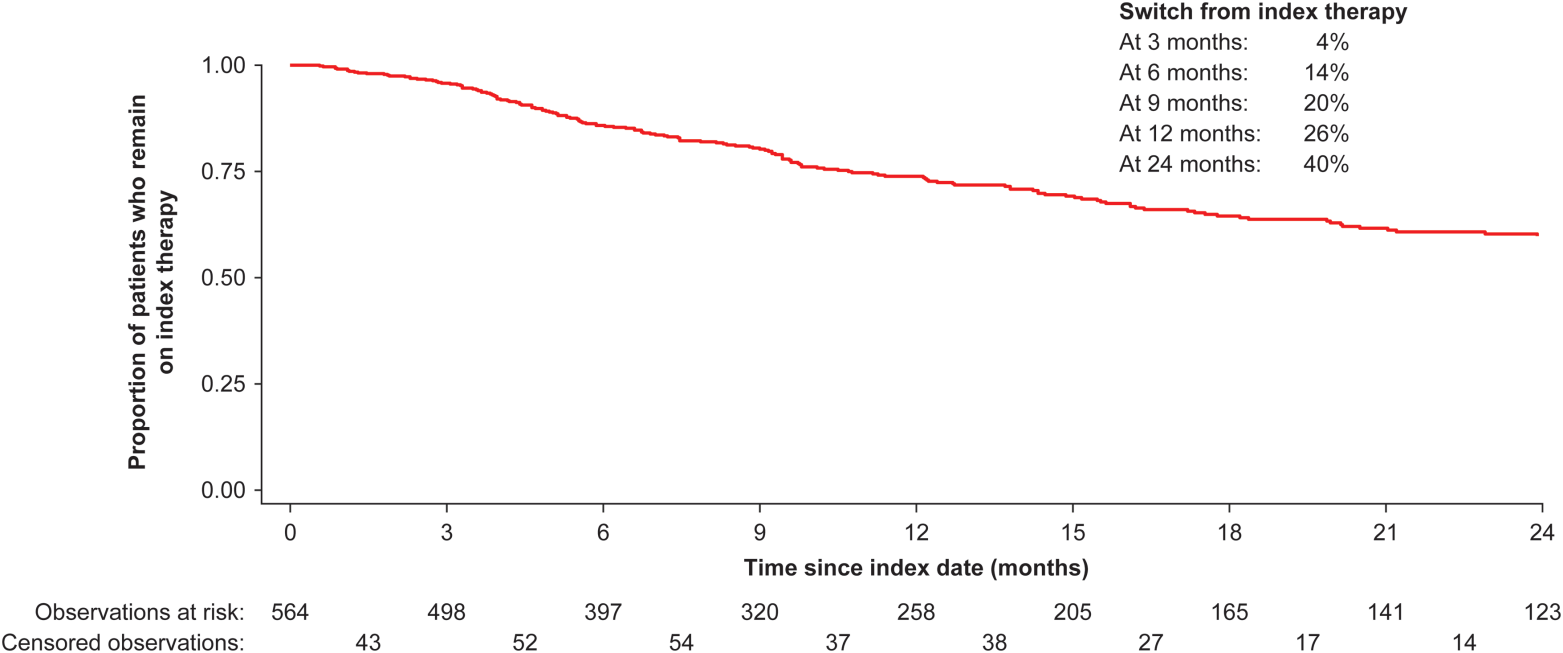
Time to first switch among patients with UC treated with advanced therapy.

There were 282 patients that discontinued their index therapy during follow-up, of which 172 patients switched from their index therapy to another advanced therapy. At 12 months and 24 months, the rates of switching from the index therapy to another advanced therapy were 26% and 40%, respectively (Figure 3). At index, the majority of patients with UC were on advanced therapy with adalimumab (*n* = 230), followed by infliximab (*n* = 172), vedolizumab (*n* = 113), golimumab (*n *= 56), and tofacitinib (*n* = 3). At the end of follow-up (median follow-up time was 26.0 months with IQR of 7.5-17.5), 145 patients were on therapy with adalimumab, of which 35 patients had switched to this agent from a different index therapy (Supplemental Figure 4). Less than half of patients remained on initial therapy when receiving adalimumab (48%), infliximab (48%), or golimumab (43%). The rate of treatment maintenance was slightly higher in patients on vedolizumab (56%) or tofacitinib (66%). At the end of follow-up, the number of patients on therapy with infliximab, golimumab, vedolizumab, and tofacitinib equaled 132, 45, 123, and 9 patients respectively. Of these, 49 patients switched from their index therapy to infliximab, 21 patients switched to golimumab, 60 patients switched to vedolizumab, and 7 patients switched to tofacitinib.

### Health Care Resource Utilization Outcomes After Therapy Initiation

Within the study cohort, 210 patients (36.6%) experienced at least 1 all-cause hospitalization with a mean length of stay (LOS) of 9.7 days, and 155 (27.0%) had at least 1 UC-related hospitalization with a mean LOS of 11.2 inpatient days. On average, gastroenterologists (GI) were visited 2.1 times per patient year; we observed that 61.2% of patients had at least 1 GI visit, but 45.3% saw their GI at least twice. Patients reported 26.3 days per patient year on sick leave; of these, 13.1 days were UC-related (Table 3).

**Table 3.** All-cause and UC-related HCRU among patients with escalation or switch of index therapy.

Type of Resource Utilization (per patient year)	Overall	Escalation of Index Therapy			Switching of Index Therapy to Another Agent		
		With Dose Escalation	Without Dose Escalation	*P* ∗	With Switch	Without Switch	*P* ∗
**Total observable patients/ patient years**	574/ 447.0	64/ 62.3	264/ 256.2		172/ 134.8	402/ 312.2	64/ 62.3
**All-cause HCRU**							
**Hospitalizations**							
Patients with hospitalizations	210 (37%)	21 (33%)	86 (33%)	0.971	79 (46%)	131 (33%)	0.002
Number of hospitalization days	9.7	6.8	6.4	0.900	14.7	7.6	<0.001
**Outpatient visits**							
Number of GP visits	3.5	4.1	3.5	0.009	3.8	3.4	0.007
Number of gastroenterologists’ visits	2.1	2.1	2.3	0.361	2.3	2.0	0.080
Number of other specialist visits	9.4	9.3	9.7	0.312	10.2	9.1	0.014
**Sick Leave days**							
Number of days	26.3	10.5	22.5	0.203	34.6	22.7	0.771
**UC-related HCRU**							
**Hospitalizations**							
Patients with hospitalizations	155 (27%)	15 (23%)	59 (22%)	0.852	63 (37%)	92 (23%)	0.001
Number of hospitalization days	11.2	4.5	4.0	0.846	10.6	5.2	<0.001
**Sick Leave days**							
Number of days	13.1	5.9	9.3	0.786	16.6	9.6	0.008
**Surgeries**							
IPAA	0.06	0.02	0.03	0.723	0.07	0.06	0.417
Rectal procedure	0.09	0.05	0.04	0.300	0.12	0.07	0.257
Anal procedure	0.00	0.00	0.00	0.623	0.01	0.00	0.536
Other	0.02	0.00	0.02	0.487	0.01	0.02	0.478

Shows all-cause and UC-specific HCRU in the overall sample of patients initiating an advanced therapy for management of UC as well as of predefined subsamples with and without therapy escalation/switch. Health care costs are reported in Euros as rate per person-year. Frequencies are reported as rate per person-year. Abbreviations: CS, corticosteroids; GP, general practitioner; HCRU, health care resource utilization; IPAA, ileal pouch anal anastomosis; UC, ulcerative colitis.

Differences between subgroups were assessed by Welch *t* test for continuous variables.

Compared with patients who maintained their index therapy, patients with at least 1 therapy switch had more frequent visits to GPs (3.4 vs 3.8 visits per patient year, *P* = .007), gastroenterologists (2.0 vs 2.3 visits per patient year, *P* = .080), and other specialists (9.3 vs 10.2 visits per patient year, *P* = .014). In addition, the number of days spent in hospital was significantly higher in patients who switched their index therapy (14.7 vs 7.6 days per patient year, *P* < .001). Total all-cause costs were higher among patients with switch of advanced compared with patients who maintained their index therapy (€44 570 vs €36 807 per patient year, *P* < .001), with higher average costs recorded for switchers in each examined cost subcategory (Table 4).

**Table 4.** All-cause and UC-related health care costs among patients with escalation or switch of index therapy.

Type of Cost	Overall	Escalation of Index Therapy			Switching of Index Therapy To Another Agent		
		With Dose Escalation	Without Dose Escalation	*P* ∗	With Switch	Without Switch	*P* ∗
**Total observable patients/ patient years**	574/ 447.0	64/ 62.3	264/ 256.2		172/ 134.8	402/ 312.2	
**All-cause costs**							
Cost for outpatient care	€1104.70	€1138.52	€1109.55	0.714	€1223.04	€1053.58	0.004
Cost for GP visits	€351.07	€410.96	€344.75	0.011	€384.15	€336.78	0.004
Cost for gastroenterologists’ visits	€185.00	€188.11	€201.23	0.613	€202.69	€177.36	0.120
Cost for other specialist visits	€568.63	€539.43	€563.56	0.721	€636.20	€539.44	0.071
Cost for medication	€30,200.93	€38,312.86	€28,945.72	<0.001	€32,908.50	€29,031.53	<0.001
Cost for inpatient care	€4522.04	€2143.68	€2,882.60	0.319	€6,202.58	€3796.22	0.027
Cost for aids and remedies	€342.42	€173.69	€318.42	0.116	€337.97	€344.35	0.951
Indirect cost (based on sick leave days)	€2978.58	€1179.93	€2,548.60	0.006	€3898.30	€2581.35	0.131
Total direct and indirect all-cause costs	€39,148.67	€42,948.67	€35,804.89	<0.001	€44,570.39	€36,807.03	< 0.001
**UC-related costs**							
Cost for outpatient care	€510.71	€524.16	€502.99	0.706	€573.45	€483.61	0.025
Cost for GP visits	€251.22	€287.64	€248.31	0.157	€268.27	€243.86	0.222
Cost for gastroenterologists’ visits	€123.47	€116.85	€133.35	0.458	€143.46	€114.83	0.064
Cost for other specialist visits	€136.02	€119.66	€121.33	0.961	€161.72	€124.92	0.153
Cost for medication	€28,885.18	€37,368.59	€28,065.99	<0.001	€31,861.56	€27,599.67	<0.001
Cost for inpatient care	€3190.50	€1620.81	€1856.31	0.726	€4109.36	€2793.64	0.108
Indirect cost (based on sick leave days)	€1481.97	€659.42	€1062.72	0.300	€2417.40	€1077.95	0.035
Total direct and indirect UC-related costs	€34,068.35	€40,172.98	€31,488.02	<0.001	€38,961.77	€31,954.88	<0.001

Shows all-cause and UC-specific costs in the overall sample of patients initiating an advanced therapy for management of UC and of predefined subsamples with and without therapy escalation/switch. Health care costs are reported in Euros as ratio of total costs assessed during study follow-up divided by total number of patient years observed. Abbreviations: CS, corticosteroids; GP, general practitioner; UC, ulcerative colitis.

Differences between subgroups were assessed using the bootstrap method for asymmetrically distribution of cost ratios.

Patients with dose escalation during the maintenance phase frequently visited GPs (4.1 vs 3.5 visits, *P* = .009; Table 3). No substantial differences were found for hospitalization days (6.8 vs 6.4 days per patient year; *P* = .920), visits to gastroenterologists (2.1 vs 2.3, *P* = .361), and other specialist visits (9.3 vs 9.7, *P* = .312).

### Health Care Cost After Therapy Initiation

The overall all-cause costs of patients with UC were €39 148.67 per patient year (Table 4). Total costs were mainly driven by outpatient medication costs with €30 200.93 per patient year, the majority of which was UC-related medication (28 885.18€ per patient year).

Total all-cause costs were higher among patients who escalated their index therapy compared with those without escalation (€42 949 vs €35 805 per patient year, *P* < .001). The same held true for total UC-related costs (€40 173 vs €31 488 per patient year, *P* < .001) due to high spending on UC-related medications.

### Adverse Events Requiring Hospitalization

There were 87 SAEs observed during the induction phase, corresponding to 0.63 (95% CI, 0.55-0.71) SAEs per person-year (Supplemental Table 2). For the maintenance phase, 101 SAEs were identified, corresponding to an event rate of 0.24 (95% CI, 0.21-0.29) per person-year. Overall, the most frequently observed AEs were anemia (*n* = 54) and severe infections (*n* = 52).

## Discussion

To the best of our knowledge, this is the first real-world study from Germany to provide data on inadequate response to advanced therapies along with their health care resource utilization (HCRU) and associated costs. Various indicators based on drug prescriptions, surgical procedures, and hospitalizations were used to determine inadequate response. We found that the vast majority of patients on advanced therapies for UC management experienced an inadequate response. Those with prior UC-related hospitalizations and concurrent use of CS at index were more likely to experience inadequate response. Half of the patients experienced their first inadequate response within 5 months. Patients often discontinued their advanced therapy and switched to other therapies. Patients with or without previous experience to biologics had similar rates of inadequate response, but those who were treated with more than 2 biologics had a higher risk of inadequate response.

A recently published German claims data analysis showed that 60% to 70% of patients were persistent users of biologic therapies at 1 year of follow-up.^9^ This is in line with our finding of 37.7% of patients discontinuing their therapy at 1 year of follow-up. Conversely, a recent US database study demonstrated that only 44.8% of UC patients were still on their initial biological therapy with either infliximab, adalimumab, certolizumab, golimumab, or vedolizumab at 1 year of follow-up.^14^ Here, discontinuation was defined as a drug-free period greater than the days of supply of the previous administration; in the current study, discontinuation was defined based on a gap of more than 60 days after the estimated consumption of the previous prescription.

Several US claims data studies have been conducted that evaluated therapy discontinuations and other indicators of suboptimal therapy in the IBD population, which includes UC. Similar to this study, Patel et al found that 51% of UC patients treated with adalimumab or infliximab had an indicator of suboptimal therapy at 6 months of follow-up, whereby treatment discontinuation of the index biologic therapy was the main indicator of suboptimal therapy.^11^ Another retrospective analysis of US claims data found that 81% of the UC patients had an indicator for suboptimal therapy, thus indicating that augmentation was common for UC patients treated with adalimumab and infliximab.^15^ These findings are in line with our current study, which found an inadequate response in 85% of patients despite the approval of novel therapies such as vedolizumab and tofacitinib. Similarly, a multinational chart review carried out in Europe and Canada found that 64.1% of UC patients naïve to anti-TNF therapy (infliximab and adalimumab) had an indicator of a suboptimal therapy within 2 years of treatment initiation. Of the UC patients that discontinued therapy, 49.5% had switched to another anti-TNF therapy.^16^ The definition of a suboptimal therapy did not include UC-related hospitalization, which was a common indicator of inadequate response in the current study (35.7% of the UC patients were hospitalized due to UC at 2 years of follow-up). Targownik et al showed that 61.3% of UC patients experienced dose augmentation using routinely collected health care utilization data from Canada.^14^ Here, dose augmentation was defined as an increase in the dose of >50% or <50 days between 2 prescriptions. The current study defined dose escalation as a dose increase of >50% over 3 consecutive prescription intervals and found that only 18.8% of UC patients experienced dose-escalation at 1 year of follow-up. Overall, the results of our study describing treatment patterns and inadequate response of advanced therapy in UC patients lay within the range of results published in the existing literature. The definition of an inadequate response and the indicators included may explain differences in estimates of suboptimal therapy observed across studies. Two German claims data studies investigated inadequate response to advanced therapy in IBD patients and showed that even after the start of biological therapy, there was still residual disease that required treatment with concomitant CS use.^9,10^

Given the limited treatment options, the possibility of dose escalation often represents the most appropriate medically reasonable alternative. In our study, 1 in 5 patients (19%) experienced a dose escalation within the first year after index therapy initiation, and 30% were augmented with a conventional therapy. Furthermore, a high proportion of patients (44%) needed prolonged use of CS. Similar results have been previously reported by Rubin et al^15^ who showed that dose escalation was common for biological therapies (17% to 35%) in UC patients. Among those who initiated anti-TNF therapy, every second patient was augmented with corticosteroids (43% to 57%). These treatment adjustments were associated with a substantial direct and indirect economic burden for payers with an approximate 0.7-fold and 1.3-fold increase in all-cause and UC-related costs.

Analysis of HCRU and costs revealed that UC-related medication was the main cost driver in patients with UC. Therefore, the highest costs were observed among patients with inadequate response to the index therapy, indicated by a dose escalation or a switch to another advanced therapy. Only 0.4% of the total costs per patient year were caused by outpatient gastroenterologist visits. This represents a problem in the context of optimized IBD care in gastroenterology practice. For adapted optimized care of patients with moderate to severe UC, €123.47 is an insufficient fee for disease-related costs per year for a biologics patient of an outpatient gastroenterologist. Increasing outpatient spending, on the other hand, could significantly improve patient management and ultimately even reduce overall treatment costs due to closer follow-up and a strengthening of treatment adherence through shared decision-making between gastroenterologists and patients.

We have shown that the bulk of cost of UC patients in Germany was driven by medications, whereas outpatient visits only contributed marginally to the total cost of UC (0.4% of UC-related costs). This depicts a different picture compared with other countries such as the United States. A US claims study in patients with/without suboptimal treatment for UC showed that IBD-related costs were driven by prescription drugs (70%/65%) and also outpatient/ambulatory encounters (30%/34%).^15^ Therefore, a meaningful interpretation of the study results in terms of economic outcomes can only be made for the German health care system.

Strengths of the current study include the focus on UC patients rather than the overall IBD patient population. In addition, the study comprised a wide range of advanced treatments targeted for UC including newer agents such as tofacitinib, which is lacking in-depth investigation on inadequate responses, as it had only been approved for the treatment of UC patients in Germany for approximately 27 months prior to the end of the study inclusion period. Furthermore, this study was based on a large claims database of approximately 3.4 million individuals for initial data extraction containing complete and detailed information on prescriptions, outpatient and inpatient diagnoses/treatment, and related costs. This information is available at the patient level and can therefore be assigned to individual treatment cases.

There are some limitations of this retrospective claims data analysis that need to be acknowledged. Clinical information concerning disease severity and activity and laboratory values are not captured in claims data, and the quality of coding of diagnoses in outpatient and inpatient setting may vary. In this study, we addressed limitations of coding quality by setting specific diagnosis requirements for patients to be considered as having UC. In addition, measures for inadequate response were approximated by clinical events or adjustment in treatment as suggested by prescription data. Therefore, the actual therapy response rate could not be specified. Also, documentation on the reasons why physicians prescribed specific treatments and dosages were not available. Furthermore, because the decision to escalate a therapy is at the physician’s discretion, some gastroenterologists might find it easier to adjust the dose if they already have more experience with the drug. Since infliximab and adalimumab were approved more than 10 years ago, there is much more experience with these therapies, which could encourage a greater willingness to escalate them.

Specific limitations for this study exist regarding the investigated therapies. Only a few UC patients had initiated therapy with tofacitinib, and inpatient OPS codes were not available for inpatient use with this agent. Therefore, no meaningful conclusions can be drawn on the use of JAK inhibitors. Furthermore, nonpersistence to use of advanced therapies might have been overestimated, since this was solely defined based on outpatient prescription and respective OPS codes in case of inpatient application as observable in German claims data. Thus, potential breaks in treatment (eg, due to stays abroad) could only be considered to a limited extent. We aimed to restrain the overestimation of treatment discontinuation by allowing for a gap period between prescriptions as part of the definition of treatment discontinuation. In addition, assumptions with regards to the prescribed dosage of the advanced therapeutic agents golimumab and vedolizumab had to be made due to the lack of detailed information in the corresponding inpatient OPS codes. During hospital stays, patients were therefore assumed to be persistent to their respective therapy.

Subgroup comparisons for HCRU outcomes and costs were not adjusted for possible differences in patient characteristics. Therefore, a causal relationship between patients who escalated or switched their index therapy and the respective cost distribution in these subgroups cannot be proven beyond doubt. In addition, the estimated costs were not adjusted for inflation, so they could potentially be biased by price fluctuations between 2015 and 2019.

Because mild to moderate adverse events not leading to a hospitalization were not available in this database, the overall tolerability of advanced therapies could not be assessed. However, the frequency of serious adverse events was approximated, inasmuch as these are well defined events that often lead to an inpatient health care encounter and are well captured in claims data, thereby minimizing the underestimation of SAEs. Anemia and severe infections requiring hospitalization were the most frequently reported SAEs. However, the relationship between an SAE and the disease or a respective active therapy could only be assumed, as causality cannot be established within a retrospective claims database study.

In conclusion, 3 out of 4 patients experienced an inadequate response to their advanced therapy in the first year of treatment, with therapy discontinuation and switch, as well as prolonged CS use being the main indicators observed. Furthermore, a substantial proportion of UC patients required hospitalization in the first year after starting an advanced therapy. The high economic burden of UC patients is mainly driven by UC-related medication cost and frequent escalation of advanced therapies. The existing body of literature on suboptimal therapy in UC patients and the current findings in this study highlight the need for additional treatment alternatives and improvements in the current daily clinical practice of UC patients that are in need of advanced therapies.

## Supplementary Material

izab330_suppl_Supplementary_Material

## Data Availability

All data relevant to this study are included in the article or uploaded as supplementary material. The source data that support the findings of this study are available from AOK PLUS, but restrictions apply to the availability of these data, which were used under license for the current study and so are not publicly available.

## References

[1] Alatab S , Sepanlou SG, Ikuta K, et al. The global, regional, and national burden of inflammatory bowel disease in 195 countries and territories, 1990–2017: a systematic analysis for the global burden of disease study 2017. Lancet Gastroenterol Hepatol. 2020;5:17–30. 31648971 10.1016/S2468-1253(19)30333-4 PMC7026709

[2] Bokemeyer B. CED-Behandlung in Deutschland: Betrachtungen zur sinnvollen Vernetzung. Gastroenterologe. 2007;2:447–455.

[3] Hou JK , Kramer JR, Richardson P, et al. The incidence and prevalence of inflammatory bowel disease among U.S. veterans: a national cohort study. Inflamm Bowel Dis. 2013;19:1059–1064. 23448789 10.1097/MIB.0b013e31828028ca PMC3972034

[4] Yarlas A , Rubin DT, Panés J, et al. Burden of ulcerative colitis on functioning and well-being: a systematic literature review of the SF-36® health survey. J Crohns Colitis. 2018;12:600–609. 29718244 10.1093/ecco-jcc/jjy024

[5] Van Assche G , Peyrin-Biroulet L, Sturm A, et al. Burden of disease and patient-reported outcomes in patients with moderate to severe ulcerative colitis in the last 12 months - multicenter European cohort study. Dig Liver Dis. 2016;48:592–600. 26935454 10.1016/j.dld.2016.01.011

[6] Torres J , Bonovas S, Doherty G, et al. ECCO guidelines on therapeutics in Crohn’s disease: medical treatment. J Crohns Colitis. 2020;14:4–22. 31711158 10.1093/ecco-jcc/jjz180

[7] Kucharzik T , Dignass AU, Atreya R, et al. Updated S3-guideline ulcerative ColitisGerman society for digestive and metabolic diseases (DGVS). Z Gastroenterol. 2019;57:162–241. 30654406 10.1055/a-0824-0861

[8] Harbord M , Eliakim R, Bettenworth D, et al. Third European evidence-based consensus on diagnosis and management of ulcerative colitis. Part 2: current management. J Crohn’s Colitis. 2017;11:769–784. 28513805 10.1093/ecco-jcc/jjx009

[9] Mevius A , Brandes A, Hardtstock F, et al. Persistence with biologic treatment in patients with inflammatory bowel disease: a German claims data analysis. Digestion. 2019. DOI: 10.1159/000503859. 31639807

[10] Brandes A , Groth A, Gottschalk F, et al. Real-world biologic treatment and associated cost in patients with inflammatory bowel disease. Z Gastroenterol. 2019;57:843–851. 31288280 10.1055/a-0903-2938

[11] Patel H , Lissoos T, Rubin DT. Indicators of suboptimal biologic therapy over time in patients with ulcerative colitis and Crohn’s disease in the United States. Plos One. 2017;12:e0175099. 28426675 10.1371/journal.pone.0175099 PMC5398513

[12] Volkswirtschaftliche Kosten durch Arbeitsunfähigkeit 2019. 2019, Accessed April 15, 2021, https://www.baua.de/DE/Themen/Arbeitswelt-und-Arbeitsschutz-im-Wandel/Arbeitsweltberichterstattung/Kosten-der-AU/pdf/Kosten-2019.pdf?__blob=publicationFile&v=3

[13] Sundararajan V , Henderson T, Perry C, et al. New ICD-10 version of the Charlson comorbidity index predicted in-hospital mortality. J Clin Epidemiol. 2004;57:1288–1294. 15617955 10.1016/j.jclinepi.2004.03.012

[14] Chen C , Hartzema AG, Xiao H, et al. Real-world pattern of biologic use in patients with inflammatory bowel disease: treatment persistence, switching, and importance of concurrent immunosuppressive therapy. Inflamm Bowel Dis. 2019;25:1417–1427. 30839057 10.1093/ibd/izz001

[15] Rubin DT , Mody R, Davis KL, Wang CC. Real-world assessment of therapy changes, suboptimal treatment and associated costs in patients with ulcerative colitis or Crohn’s disease. Aliment Pharmacol Ther. 2014;39:1143–1155. 24697826 10.1111/apt.12727

[16] Lindsay JO , Armuzzi A, Gisbert JP, et al. Indicators of suboptimal tumor necrosis factor antagonist therapy in inflammatory bowel disease. Dig Liver Dis. 2017;49:1086–1091. 28826571 10.1016/j.dld.2017.07.010

