# Supplementary material for: Inadequate Response, Treatment Patterns, Health Care Utilization, and Associated Costs in Patients With Ulcerative Colitis: Retrospective Cohort Study Based on German Claims Data
Source: Inflamm Bowel Dis. 2022 Feb 4;28(11):1647–57. doi: 10.1093/ibd/izab330 (PMC9629453; doi:10.1093/ibd/izab330)
Supplement: izab330_suppl_Supplementary_Material [file izab330_suppl_supplementary_material.docx]

# Supplemental materials

Supplemental Table 1: List of ICD-10, ATC, OPS, and EBM codes for identification of advanced therapeutic agents, exclusion criteria, corticosteroids, other conventional therapies, UC-related surgeries, UC-related complications.

| Code | Description | Coding system | Grouping |
| --- | --- | --- | --- |
| M05.0 –M05.9; M06.0 | Rheumatoid arthritis | ICD-10 | Exclusion criteria |
| K50 | Crohn’s disease | ICD-10 | Exclusion criteria |
| K52.3 | Colitis indeterminate | ICD-10 | Exclusion criteria |
| L40.- | Psoriasis | ICD-10 | Exclusion criteria |
| G35.- | Multiple sclerosis | ICD-10 | Exclusion criteria |
| M45.- | Axial spondylarthritis/Ankylosing spondylitis | ICD-10 | Exclusion criteria |
| L40.- | Psoriatic arthritis | ICD-10 | Exclusion criteria |
| L73.2 | Hidradenitis suppurativa/Acne inversa | ICD-10 | Exclusion criteria |
| H20.-, H30.- | Uveitis intermedia, uveitis posterior and panuveitis | ICD-10 | Exclusion criteria |
| 5-45- | Intestinal incision, excision, resection, and anastomosis | OPS | UC-related surgeries |
| 5-46- | Other intestinal procedures | OPS | UC-related surgeries |
| 5-48- | Rectal procedures | OPS | UC-related surgeries |
| 5-49- | Anus procedures | OPS | UC-related surgeries |
| 31157, 36157 | Intestinal incision, excision, resection, and anastomosis | EBM | UC-related surgeries |
| 31154, 36154 | Other intestinal procedures | EBM | UC-related surgeries |
| 31175, 36175 | Rectal procedures | EBM | UC-related surgeries |
| 31172, 36172 | Anal procedures | EBM | UC-related surgeries |
| H02AB-; H02B- | Corticosteroids | ATC | CS for systemic use |
| A07EA01 | Prednisolone | ATC | Locally acting CS |
| A07EA02 | Hydrocortisone | ATC | Locally acting CS |
| A07EA03 | Prednisone | ATC | Locally acting CS |
| A07EA04 | Betamethasone | ATC | Locally acting CS |
| A07EA05 | Tixocortol | ATC | Locally acting CS |
| A07EA06 | Budesonide | ATC | Locally acting CS |
| A07EA07 | Beclomethasone | ATC | Locally acting CS |
| A07EA51 | Prednisolone (combination) | ATC | Locally acting CS |
| K61.- | Abscesses | ICD-10 | UC-related complications |
| H20.0 | Acute and subacute iridocyclitis | ICD-10 | UC-related complications |
| K83.0 | Cholangitis | ICD-10 | UC-related complications |
| L52.- | Erythema nodosum | ICD-10 | UC-related complications |
| L88.- | Pyoderma gangraenosum | ICD-10 | UC-related complications |
| K83.- | Primary Sclerosing Cholangitis | ICD-10 | UC-related complications |
| L04AB02 | Infliximab | ATC | Advanced therapy for UC |
| L0SAB04 | Adalimumab | ATC | Advanced therapy for UC |
| L04AB06 | Golimumab | ATC | Advanced therapy for UC |
| L04AA33 | Vedolizumab | ATC | Advanced therapy for UC |
| L04AA29 | Tofacitinib | ATC | Advanced therapy for UC |
| L04AX01 | Azathioprine | ATC | UC-associated conventional therapy |
| L01BB02 | Mercaptopurine | ATC | UC-associated conventional therapy |
| L01BA01, L04AX03 | Methotrexate | ATC | UC-associated conventional therapy |
| H02- | Corticosteroids for systemic use | ATC | UC-associated conventional therapy |
| A07EA | Corticosteroids acting locally | ATC | UC-associated conventional therapy |
| A07EC | 5-aminosalicylic acid | ATC | UC-associated conventional therapy |
| A07A-, J01- | Antibiotics | ATC | UC-associated conventional therapy |
| L04AD01 | Ciclosporin | ATC | UC-associated conventional therapy |
| L04AD02 | Tacrolimus | ATC | UC-associated conventional therapy |

Supplemental Table 2: Number of SAEs per person-year (95%-CI) reported for the induction and maintenance period

|  | Induction period | | Maintenance period | |
| --- | --- | --- | --- | --- |
| *Type of SAE* | *Number of events* | *Event rate per person-year (CI)* | *Number of events* | *Event rate per person-year (CI)* |
| Composite (any UC-related SAE) | 87 | 0.63 (0.55-0.71) | 101 | 0.24 (0.21-0.29) |
| Anemia | 34 | 0.25 (0.18-0.33) | 20 | 0.05 (0.03-0.07) |
| Primary infections (requiring hospitalization)^1^ | 26 | 0.19 (0.13-0.26) | 26 | 0.06 (0.04-0.09) |
| Opportunistic infections^2^ | 12 | 0.09 (0.05-0.15) | 12 | 0.03 (0.02-0.05) |
| Skin conditions^3^ | 11 | 0.08 (0.04-0.14) | 10 | 0.02 (0.01-0.04) |
| Abscess of anal and rectal regions | 9 | 0.07 (0.03-0.12) | 11 | 0.03 (0.01-0.05) |

SAE event rates are reported as per person-year (95%-CI), separately for occurrence within the induction period and maintenance period. *SAE*, serious adverse event.

1) Primary infections were defined as any diagnosis of infectious diseases resulting from primary pathogens in inpatient setting requiring 3 days of hospitalization

2) Opportunistic infections include tuberculosis, CMV, fungal and parasitic infections

3) Skin conditions include skin ulcer, stomatitis, anal fissures, and other intestinal fistulas

*CI*, confidence interval; *SAE*, serious adverse event

Supplemental Figure 1: Selection of study cohort


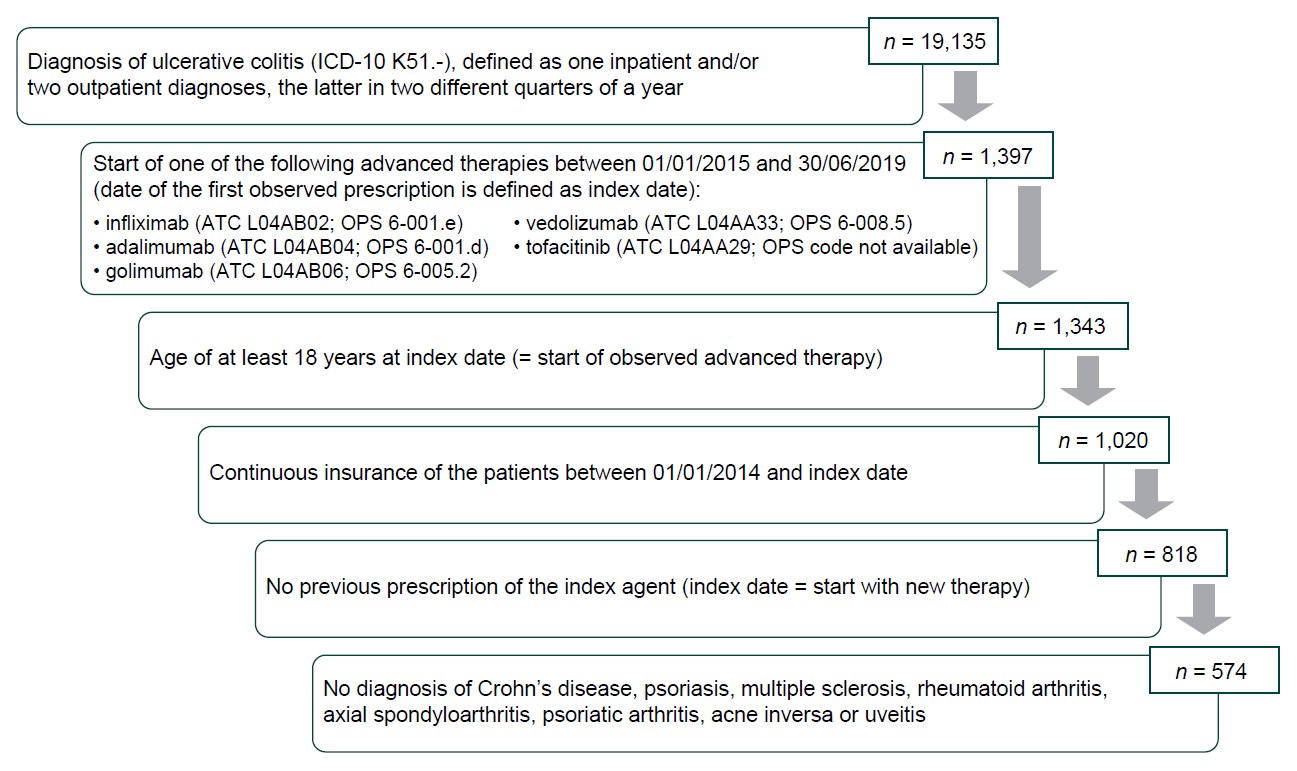


Supplemental Figure 2: Attrition chart for definition of the study cohort


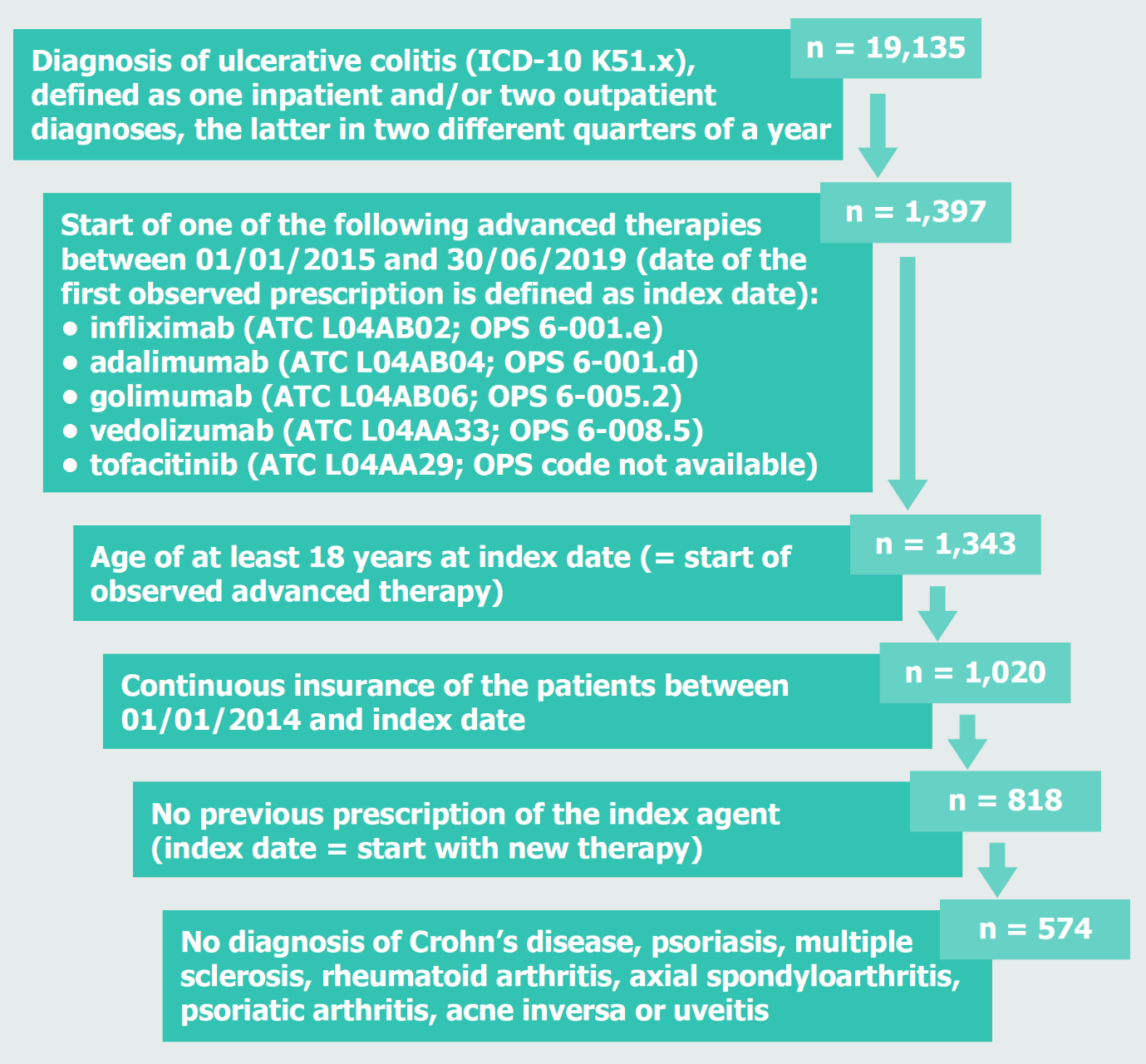


Supplemental Figure 3: Time to advanced therapy discontinuation among patients with previous exposure to biologics


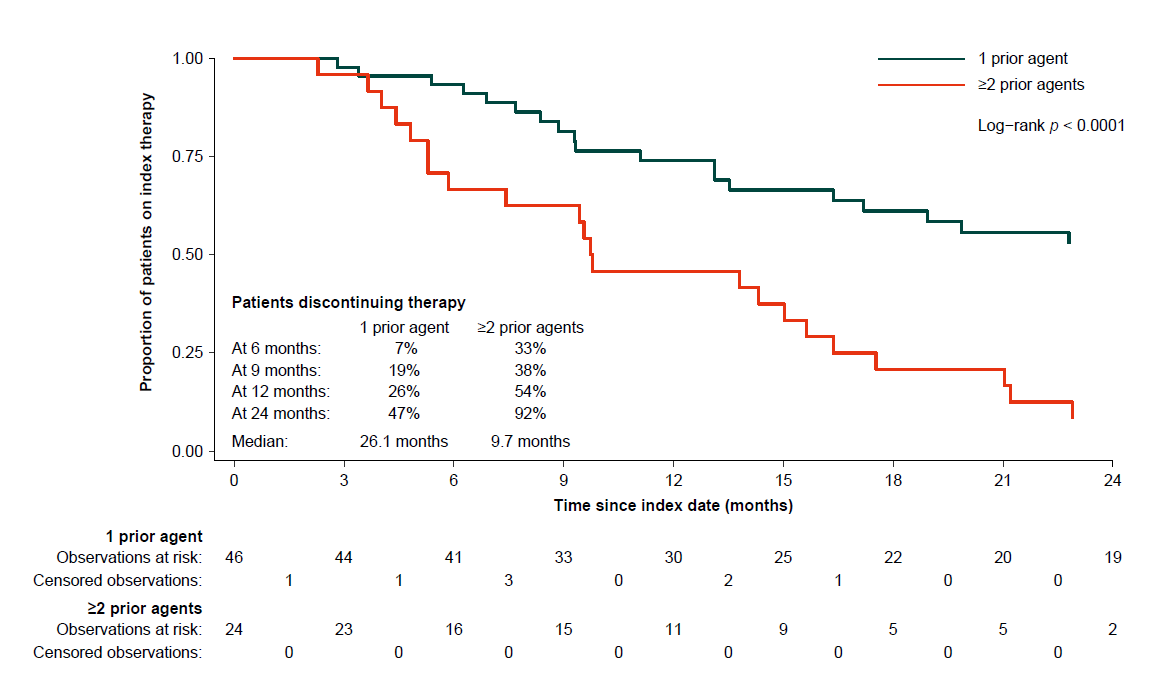


Kaplan-Meier-curve showing time to advanced therapy discontinuation for UC patients with a history of 1 advanced therapy prior to the index therapy and UC patients with a history of ≥2 advanced therapies prior to the index therapy.

Supplemental Figure 4: **Treatment pathwaya among patients with UC treated with advanced therapy**


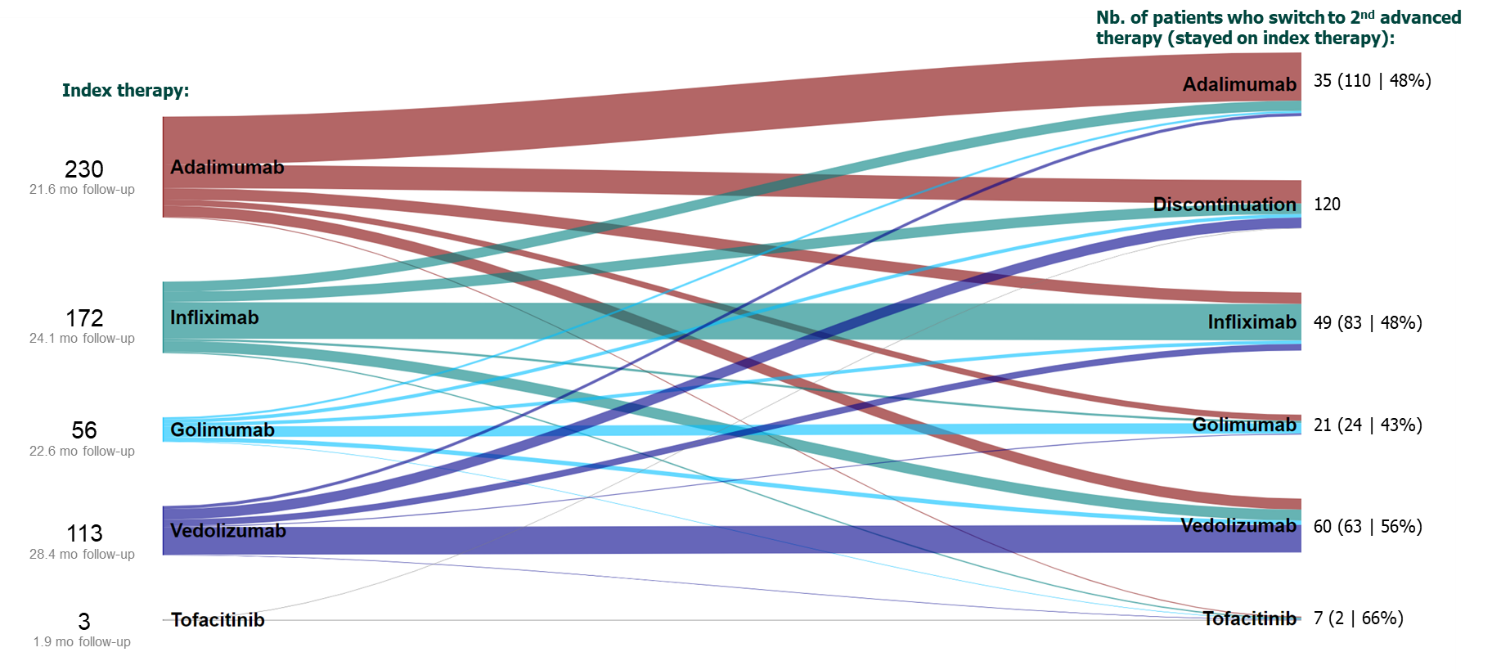


*UC, ulcerative colitis*
